# Free Faecal Water: Analysis of Horse Faecal Microbiota and the Impact of Faecal Microbial Transplantation on Symptom Severity

**DOI:** 10.3390/ani11102776

**Published:** 2021-09-23

**Authors:** Louise Laustsen, Joan E. Edwards, Gerben D. A. Hermes, Nanna Lúthersson, David A. van Doorn, Supattra Okrathok, Theresa J. Kujawa, Hauke Smidt

**Affiliations:** 1 Department of Veterinary Education, Institute of Veterinary Science, University of Liverpool, Neston CH64 7TE, UK; vetlouiselaustsen@gmail.com; 2 Laboratory of Microbiology, Wageningen University & Research, 6708 WE Wageningen, The Netherlands; gerben.hermes@wur.nl (G.D.A.H.); s.okrathok@gmail.com (S.O.); 3 Hestedoktoren, 4360 Kirke Eskilstrup, Denmark; nanna@hestedoktoren.dk; 4 Department of Clinical Sciences, Equine Health, Utrecht University, 3584 CM Utrecht, The Netherlands; d.a.vandoorn1@uu.nl; 5 Department Population Health Sciences, Farm Animal Health, Utrecht University, 3584 CL Utrecht, The Netherlands; theresa.k2k@gmail.com; 6 School of Animal Technology and Innovation, Institute of Agricultural Technology, Suranaree University of Technology, Nakhon Ratchasima 30000, Thailand; 7 Animal Nutrition Group, Wageningen University & Research, 6708 WE Wageningen, The Netherlands

**Keywords:** bacteria, archaea, prokaryote, equine, hindgut, faeces, faecal water syndrome, free faecal liquid

## Abstract

**Simple Summary:**

Free faecal water (FFW) in equines causes soiling of the hindquarters and tail and may also include additional symptoms. The cause of FFW is unknown. In this study it was investigated whether the microbes present in the last part of the gut (i.e., the hindgut microbiota) may be involved. From the analysis of faecal samples, it was found that horses suffering from FFW had no differences in their hindgut microbiota compared to healthy horses stabled at the same location. However, subsequent treatment of the FFW horses with a faecal microbiota transplantation (FMT) from a healthy donor animal resulted in a decrease in FFW symptom severity. Nevertheless, animals did not respond uniformly to this treatment, with some only having temporary decreases in FFW symptom severity. No lasting changes in the hindgut microbiota of the FFW horses occurred as a result of the faecal transplant. The practical implication of these findings is that FMT can potentially be used to temporarily alleviate FFW symptom severity in horses, although future studies using controls are needed to confirm the effectiveness of FMT to treat FFW.

**Abstract:**

Free faecal water (FFW) in equines results in pollution of the hindquarters and tail and can also involve clinical signs. Though the cause of FFW is unknown, it was hypothesized that it may involve the gut microbiota. This hypothesis was addressed as follows. First, the faecal prokaryotic community composition of horses suffering from FFW relative to healthy controls (*n* = 10) was compared. Second, FFW horses were treated with a standardised faecal microbiota transplantation (FMT) protocol (*n* = 10), followed by assessment of FFW symptom severity and faecal prokaryotic community composition over a follow-up period of 168 days. No significant differences were found in the faecal microbiota composition of FFW horses compared to healthy controls (*p* > 0.05). Relative to before FMT, FFW symptom severity decreased in affected horses 14 days after FMT (*p* = 0.02) and remained decreased for the remainder of the study (*p* < 0.02). However, individual animal responses to FMT varied. FMT had no effect on FFW horse faecal prokaryotic community composition in terms of alpha or beta diversity. Alpha diversity of the donor inocula used in the FMT was always lower than that of the faecal microbiota of the FFW treated horses (*p* < 0.001). In conclusion, whilst findings indicate FFW horses do not have an altered hindgut microbiota, some horses that received FMT had a temporary alleviation of FFW symptom severity without causing changes in the faecal microbiota. Future studies using controls are now needed to confirm the effectiveness of FMT to treat FFW.

## 1. Introduction

Free faecal water (FFW), also known as faecal water syndrome or free faecal liquid, results in pollution of the hindquarters and tail. It has been indicated that only subtle changes occur in the general health of affected animals [1]. However, recently in a more extensive survey 65% of the FFW horses were reported to have clinical signs [2]. These signs included colic, irritation when voiding faeces and a bloated abdomen. Horses suffering from FFW also had a higher incidence of colic than the general horse population, and a previous history of colic was associated with clinical signs occurring during FFW episodes [2]. The cause of FFW is not currently known. Factors that have been previously suggested to play a role in FFW development include social stress, diet and low-grade gut inflammation [1,2,3].

Whilst there is increasing evidence of a relationship with stress and the gut microbiota-brain axis [4], stress is not a sole explanation for the occurrence of FFW [2,5]. Diet has a strong influence on equine hindgut microbiota as well as digestion and digestive health [6,7,8]. Imbalances in the hindgut microbiota are often associated with gut-mediated disease and gut inflammation [9,10,11,12,13]. The role of the gut microbiota as a factor in FFW development has been previously speculated on [3]. Recently two studies assessed the faecal microbiota of horses suffering from FFW [5,14]. In both studies there was no indication of any gut microbiota disruption and, within sampling periods, differences in certain minor taxa were detected in FFW horses relative to healthy horses [5,14]. However, both studies had methodological limitations. The storage condition of collected faecal samples before analysis was not optimal (i.e., 2 weeks at 4 °C [5] or up to 3 days at ambient temperature [14]). Furthermore, the culture-independent microbiota analysis methods failed to detect the third most predominant bacterial phylum in the equine hindgut, Kiritimatiellaeota [15,16]. The detection of Kiritimatiellaeota (formerly Verrucomicrobia subdivision 5) is particularly important as its relative abundance has been previously shown to be altered in horses treated with antibiotics and those suffering from laminitis [11,17].

Faecal samples from FFW horses have been shown to be negative for *Clostridioides difficile* (formerly called *Clostridium difficile*; *C. difficile*) and *Clostridium perfringens* (*C. perfringens*) infection [14]. However, after enrichment, twice as many FFW horse faecal samples tested positive for *C. perfringens* compared to faeces from healthy control animals [14]. Pathogenic bacteria can cause gut inflammation as well as disturb the gut microbiota. A potential treatment approach to restoring disrupted gut microbiota is faecal microbiota transplantation (FMT). This approach is based on the transfer of faecal microbiota from healthy donors into the gut of recipient patients. In human medicine, FMT is an established method to treat severe and sub-acute *C. difficile* infection. This is due to the limited efficacy of antimicrobial therapy which cannot alone restore the associated gut disturbances. FMT has also received interest in terms of ulcerative colitis [18], metabolic syndrome [19], irritable bowel syndrome [20,21] and, more recently, restoring the gut microbiota development of babies born by caesarean section [22]. FMT has also a long history in veterinary practice where it has value in the treatment of various gut-related disorders in cattle, although a strong evidence base behind this is limited in terms of scientific literature [23,24,25].

Anecdotal accounts of FMT in equine medicine date back decades [26], however, information regarding its efficacy is limited [25,27]. A review on equine FMT indicated that findings to date provide initial support for its potential efficacy [25]. In a recent study, three of five diarrheic horses responded to FMT, although it should be noted that no control horses were included in the study [13]. Furthermore, as faecal microbiota analysis in this study was limited (i.e., only during and one day after FMT) it is not clear whether the immediate changes observed in the faecal microbiota of the patients persisted longer term. In humans, it has been reported that changes of the gut microbiota can persist for months [21,28].

Based on current knowledge, it was hypothesised that horses suffering from FFW have a different gut microbiota compared to healthy horses, and that symptom severity can be decreased using FMT. In order to test these hypotheses, the following was performed: (a) faecal microbiota of horses suffering from FFW (*n* = 10) and healthy control horses (*n* = 10) were compared, and in a pilot clinic field study (b) the ten FFW horses were then treated with a standardised FMT protocol. Symptom severity and faecal microbiota was assessed over a follow-up period of 168 days in the FFW horses. Study findings indicated that there was no evidence that FFW horses had an altered faecal microbiota, but that FMT can help to temporarily alleviate FFW symptom severity despite no changes in the faecal microbiota being evident.

## 2. Materials and Methods

### 2.1. Study Design and Ethical Approval

The study was conducted during October 2016 to December 2017. The study was performed within the ethical constraints governing the use of client’s horses. Written informed consent for participation in the study was obtained from all horse owners. The study is reported in two parts. In the first part, the faecal microbiota of horses suffering from FFW (*n* = 10) was compared to that of healthy control horses (*n* = 10) (Section 2.2). In the second part, the same FFW horses (*n* = 10) were then treated with a standardized FMT protocol, and the outcome of the treatment was evaluated (Section 2.3). Rectal grab sampling of faeces from all the horses involved in the study was performed following a standardized protocol that was approved by the Animal Welfare Body of Wageningen University & Research.

### 2.2. Comparison of Faecal Microbiota of FFW Horses and Healthy Control Horses

The horses suffering from FFW (*n* = 10) were all adult (i.e., >3 years of age) and had a history of FFW for >12 months. The FFW animals had no resolution of symptoms despite a diagnostic work-up by the animals’ own veterinarians and various previous treatments (Table S1). For each FFW animal, a control animal was selected from the same location. Control animals (*n* = 10) had no history of gut-related illness within the last 12 months and were matched as closely as possible to the FFW animals in terms of diet and management. The FFW animals were six adult warmblood horses (five geldings and one mare) and four adult ponies (three geldings and one mare) ranging from 6–21 years of age (average 10.5, standard deviation 5.06). Controls were six adult warmblood horses (three geldings, two mares and one stallion) and four adult ponies (three geldings and one mare) ranging from 4–17 years of age (average 10.9, standard deviation 4.20). For simplicity, ponies and horses are collectively referred to as horses. Further details of the control and FFW horses are provided in Table S2. For each FFW and control horse, a rectal grab sample of faeces was collected by hand using a new non-sterile glove for each sample. A representative subsample of the faeces was placed in a sterile tube. The faecal samples were then kept chilled using ice packs (maximum of 3 h) until they could be stored at −20 °C for later microbiota analysis.

### 2.3. Assessment of the Effect of FMT on Horses Suffering from FFW

**Figure 1:**
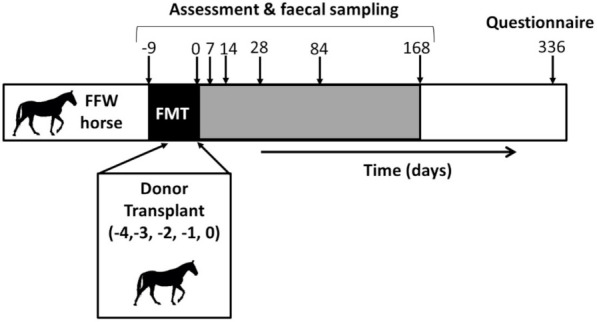
Clinical field study overview. Ten free faecal water (FFW) horses were treated with a standardized faecal microbiota transplant (FMT) protocol for 10 days (d –9 to d 0). Donor transplants were administered on five consecutive days (d –4 to d 0) of the standardized FMT protocol. After d 0, FFW horses received no further treatment. FFW horses were then visited multiple times over a 24-week period (i.e., d 7 to d 168) for post FMT assessment and faecal sampling. Faecal samples were collected before (d –9) and after (d 0, 7, 14, 28, 84 & 168) the treatment was completed. At 48 weeks (i.e., d 336) post FMT, a telephone questionnaire was performed as a final follow-up.

An overview of the clinical field study is presented in Figure 1. The same ten FFW horses described in Section 2.2 were used for the clinical field study. Throughout the study, the same veterinarian visited and assessed all the FFW horses. At the start of the clinical field study (d –9), FFW horses (*n* = 10) were visited by a trained veterinarian who collected data (see Section 2.4) and a rectal grab faecal sample from each horse (as described in Section 2.2). Directly following the d –9 visit, the FFW horses started receiving the standardized FMT protocol (Table 1) used at the clinic (Hestedoktoren, Kirke Eskilstrup, Denmark). The first part of the protocol was to receive a proton-pump inhibitor (omeprazole) for 10 days (i.e., d –9 to d 0). This was given in order to minimize inhibition of the donor inocula by the acid gastric environment [27]. The FFW horses were then given FMT for five consecutive days (d –4 to d 0) as described in Table 1.

Faecal transplants were prepared daily using freshly collected faeces from one of two donor horses (A or B) via rectal grab sampling. Donor animals were selected based on numerous criteria (Table 1). The two donors were adult warmblood mares from a herd of healthy horses stabled at the clinic of Hestedoktoren. The faecal donor horses had no history of gut disease, and no medical treatment other than standard anthelminthic treatment within the 12 months prior to the start of the study. Further details of the donor horses are provided in Table S2.

Donor A was used for six FFW horses, and donor B was used for four FFW horses. For each FFW horse, samples of the transplanted donor inocula from each day (i.e., d –4, d –3, d –2, d –1 and d 0) were stored at −20 °C for later microbiota analysis. After the final FMT was performed on d 0, a faecal sample was collected from the patient and stored at −20 °C for later microbiota analysis. After d 0, no further treatment was given to the patients.

Four follow-up visits (d 7, d 14, d 84 and d 168) of the FFW horses were conducted in the same manner as the d –9 visit. A final follow-up with FFW horse owners was also performed using a telephone-based questionnaire on d336. FFW horses were housed in the owners’ facilities throughout the study except for d –4 to d 0, when they were housed at the clinic of Hestedoktoren.

### 2.4. Data Collection

During the first visit for the clinical field study (i.e., d –9), a questionnaire was completed which documented information about the FFW horse and any other information the owner felt relevant. The questionnaire was then revisited at each subsequent visit, and any changes or additional information noted. During each of the visits, the body condition score (BCS) and FFW symptom severity was graded.

**Figure 2:**
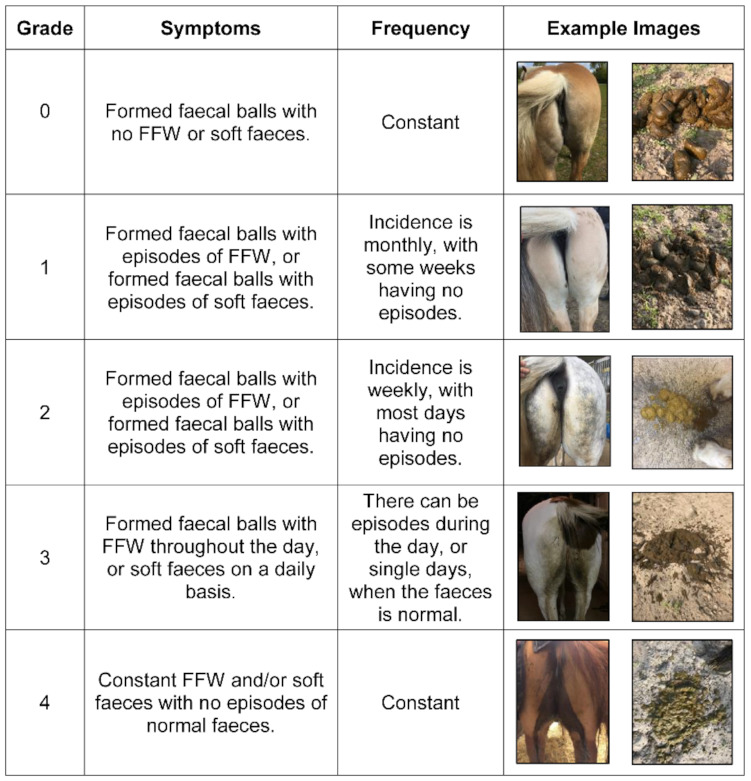
Symptom severity scale (SSS) used for grading free faecal water (FFW) symptoms in horses from 0 (no free faecal water) to 4 (maximum severity).

Body condition was scored using Kohnke’s modification [29] of the system originally described by Henneke et al. [30], and BCS ranged from 1 (very poor) to 9 (extremely fat). Grading of FFW severity was performed using a symptom severity scale (SSS) that was developed in this study (Figure 2). The SSS scale ranged from 0 (no symptoms) to 4 (maximum severity) and involved rectal grab sampling of faeces in order to evaluate the consistency of the faecal material. Furthermore, at each visit FFW horses were photographed from behind in order to give a visual impression of FFW severity. For this purpose, owners were asked not to clean the rear of the horses for 24 h before a visit.

### 2.5. DNA Extraction from Faecal Samples and Donor Inocula

Faecal samples were freeze-dried to a constant weight, and then manually ground using a mortar and pestle. The ground faecal material (25 mg) was then used to extract DNA. For donor inoculum samples, 2 mL of the inocula was centrifuged at 15,000× *g* for 5 min at 4 °C, and the resulting microbial pellet used for DNA extraction.

DNA was extracted using a MoBio PowerSoil DNA isolation kit (QIAGEN Benelux BV, Venlo, Netherlands). The manufacturer’s protocol was followed except that after the addition of buffer C1, the samples in the PowerBead tubes were processed in a bead beater (Precellys 24, Bertin technologies, Montigny-le-Bretonneux, France) for 3 × 1 min at 5.5 m/s. The resulting DNA extracts were then further purified using the Zymo Research OneStep PCR inhibitor removal kit (BaseClear Lab Products, Leiden, The Netherlands) following manufacturer’s instructions.

The purity of the resulting DNA extract was assessed using a NanoDrop ND-1000 spectrophotometer (NanoDrop^®^ Technologies, Wilmington, DE, USA), and the quantity determined using a Qubit dsDNA BR assay (Thermo Scientific, Breda, The Netherlands).

### 2.6. Prokaryotic Community Composition Profiling

Barcoded amplicons from the V4 region of prokaryotic 16S rRNA genes were generated using a barcoding strategy and primers as previously described [31]. The primer pair used was 515F (5′-GTGCCAGCMGCCGCGGTAA)—806R (5′-GGACTACHVGGGTWTCTAAT) and each primer contained an additional two base-pair linker and a custom designed eight base barcode as previously described [31]. PCR cycling conditions consisted of 98 °C for 30 s followed by 25 cycles (98 °C for 10 s, 56 °C for 10 s and 72 °C for 10 s) and then 72 °C for 7 min. Triplicate PCR reactions were prepared for each sample, along with a non-template control (NTC) reaction. The presence (samples) or absence (NTC) of PCR products was confirmed by agarose gel electrophoresis.

Pooled triplicate sample reactions were then purified using HighPrep^TM^ (MagBio Europe Ltd., Kent, UK) and quantified using a Qubit dsDNA BR Assay Kit. Purified PCR products were mixed in equimolar amounts and pooled together with defined synthetic mock communities [31] Pools then underwent adaptor ligation followed by sequencing on the Illumina HiSeq platform using 150 nucleotides paired end (PE) sequencing (GATC-Biotech, Konstanz, Germany, now part of Eurofins Genomics Germany GmbH).

The 16S rRNA gene sequence data was analysed using NG-Tax [31]. This involved the following steps being performed. Paired-end libraries were demultiplexed using read pairs with perfectly matching barcodes. Amplicon sequence variants (ASV) were picked as follows: sequences were ordered by abundance per sample and reads were considered valid when their cumulative abundance was ≥0.1%. Taxonomy was assigned using the SILVA reference database version 132 [32]. ASVs are defined as individual sequence variants rather than a cluster of sequence variants with a shared similarity above a specified threshold, such as operational taxonomic units. NG-Tax (version NG-Tax-1.jar, which is available at http://download.systemsbiology.nl/ngtax/, accessed 13 September 2019) was run with the following default settings: 70 nucleotide read length, ratio ASV abundance 2.0, classify ratio 0.8, minimum percentage threshold 0.1%, identity level 100% and error correction of one mismatch.

The raw sequence data for the first part of the study where samples from both FFW and control horses were analysed was deposited in the European Nucleotide Archive (ENA) under study accession number PRJEB35172. The raw sequence data for the longitudinal analysis of the ten FFW horses (i.e., horses P1–P10 with samples from d –9, d 0, d 7, d 14, d 28, d 64 and d 128) and the associated donor inocula they received (i.e., D-4, D-3, D-2, D-1 and D0) is deposited in the ENA under study accession number PRJEB45364.

### 2.7. Statistical Analysis

For clarity, the statistical analysis of the data is reported according to the two different parts of the study. Both univariate and multivariate data were tested for normal distribution using a Shapiro–Wilk’s test (unless indicated otherwise). For data that was not normally distributed, statistical analysis was performed using tests that did not have an underlying assumption that data was normally distributed. Analysis of the microbial data was performed with R (version 3.4.0) [33]. Significant differences were defined at *p* < 0.05.

#### 2.7.1. Comparison of Faecal Microbiota of FFW Horses and Healthy Control Horses

The sequence data was transformed from absolute counts into proportional values. Alpha diversity was calculated using four different metrics. The phylogenetic diversity metric [34] was calculated using the Picante package [35]. The metrics InvSimpson, Shannon and ASV richness were calculated using the Phyloseq package [36]. All four sets of metrics were analysed using a Student’s *t*-test. Pairwise beta diversity was calculated using weighted and unweighted UniFrac distances [37], and both matrices were visualized using principal coordinate analysis (PCoA) in the Phyloseq package [36]. The UniFrac metrics were also used to assess if the microbiota of co-located horses were more similar to each other than to other horses in the same group (i.e., did the co-location approach help to minimize background variation). This was tested using a Student’s *t*-test on within and between group distances.

To determine whether the microbiota of FFW and control horses were correlated, Procrustes analysis was performed on both ordinations using the Vegan package [38]. The protest function, which is a permutational test of the significance of the Procrustes results, was used with 999 permutations to test the significance of the correlation. In order to identify any taxa that significantly differed between the control and FFW horses, Kruskal Wallis was used to assess if any ASVs or genera were significantly affected by group (i.e., FFW or control) and resulting *p* values corrected for multiple testing using Bonferroni.

#### 2.7.2. Assessment of the Effect of FMT on Horses Suffering from FFW

The effects of FMT over time on SSS, BCS and change in BCS (relative to d –9) were analysed using repeated measure ANOVA. Normal distribution of SSS, BCS, and change in BCS relative to d –9, was visually confirmed using Q–Q plots. When ANOVA indicated significant effects (*p* < 0.05), Student’s *t*-tests were then performed between the days to confirm where significant differences occurred (Microsoft Excel).

The sequence data was transformed from absolute counts into proportional values. Bar graphs were created by summarizing the microbiota to family level and taking the top 20 families, and all other families were collected in the ‘other’ category. Alpha diversity of the prokaryotic community composition was calculated using the metrics described above. A repeated measure ANOVA was used to compare all the donor inocula samples against all the FFW horse faecal microbiota samples. Repeated measure ANOVA with a Tukey post hoc test and Bonferroni correction was also performed to look for effects of different sampling days on the FFW horse faecal microbiota.

Pairwise beta diversity was calculated using weighted and unweighted UniFrac distances (as described above). Matrices were visualized using PCoA for the complete dataset and also for subsets of the data (i.e., faecal samples from individual FFW horses). The similarity of the FFW horse faecal microbiota with the donor inocula over time was calculated by comparing all pairwise distances of a FFW horse faecal sample with all donor inocula samples e.g., faecal sample -9 was compared with donor inocula samples D-4, D-3, D-2, D-1, D0, then faecal sample d0 was compared with donor inocula sample D-4, D-3, D-2, D-1, D0 etc.). This was done for both weighted and unweighted UniFrac pairwise distances respectively. A Student’s *t*-test was used to determine *p* values.

## 3. Results

### 3.1. Comparison of Faecal Microbiota of FFW Horses and Healthy Control Horses

The average number of sequence reads per faecal sample was 226613 (standard deviation (SD) 53112). The faecal prokaryotic community of both the FFW and control horses comprised 13 different phyla. Of these phyla, the following five were most predominant (>2.5%) in both the FFW and control horses: Bacteroidetes, Firmicutes, Kiritimatiellaeota, Spirochaetes and Fibrobacteres (Table S3). The faecal prokaryotic community of FFW and control horses did not significantly differ in terms of alpha diversity when assessed using the metric phylogenetic diversity (FFW mean 16.07, SD 1.22; control mean 15.58, SD 1.34; *p* = 0.40), richness (FFW mean 239, SD 30.3; control mean 214, SD 27.2; *p* = 0.071), Shannon (FFW mean 4.9, SD 0.17; control mean 4.8, SD 0.26; *p* = 0.121) or InvSimpson (FFW mean 72.6, SD 15.92; control mean 63.5, SD 21.70; *p* = 0.300).

**Figure 3:**
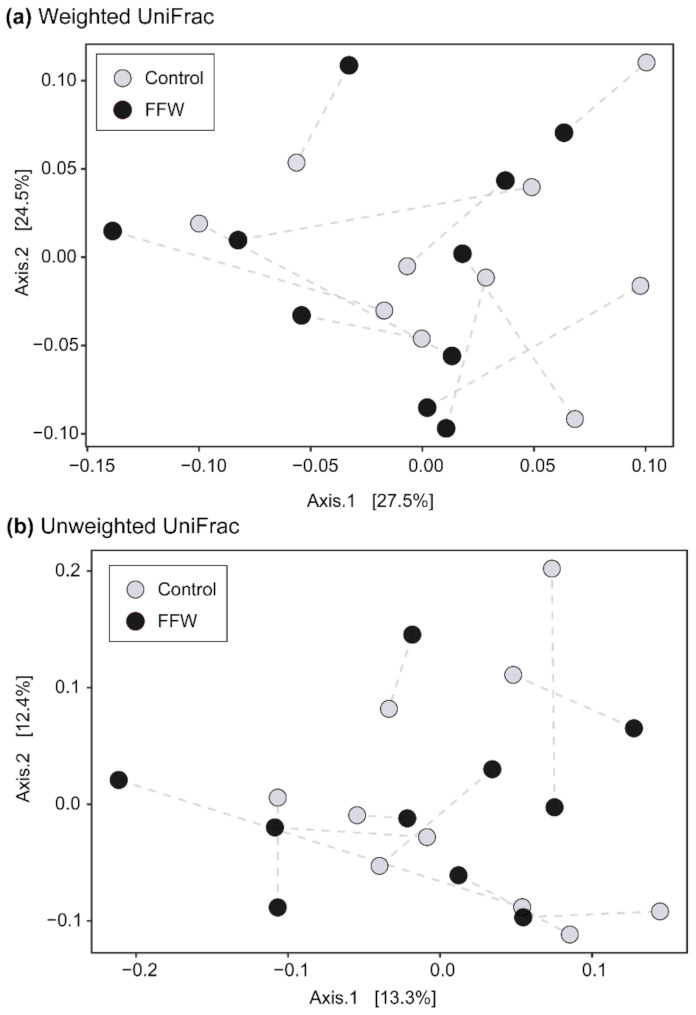
PCoA plots of 16S rRNA gene-based faecal prokaryotic community composition data from FFW and control horses using weighted (**a**) and unweighted (**b**) UniFrac distances. Co-located FFW and control horses are indicated by dashed lines.

PCoA showed no obvious clustering of FFW and control horse faecal microbiota irrespective of whether ASV relative abundance was considered (weighted UniFrac) or not (unweighted UniFrac) (Figure 3). Procrustes analysis also indicated that the beta diversity of the faecal prokaryotic community of FFW and control horses was not significantly correlated (weighted UniFrac, *p* = 0.61; unweighted UniFrac, *p* = 0.56) (Figure S1).

**Figure 4:**
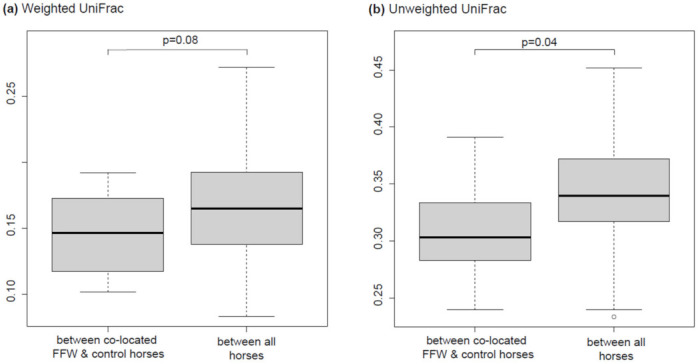
Boxplots of the weighted (**a**) and unweighted (**b**) UniFrac distances between co-located FFW and control horses and all horses are shown. A distance of 0 indicates the samples are identical, and higher values (up to a maximum of 1) indicate the extent of differences between compared samples. Boxes show the 25th and 75th percentiles with the median represented by a horizontal line. Whiskers show the data range, with the exception of one outlier, which is indicated by a data point.

Co-located FFW and control horses were more similar to each other than other horses when ASV relative abundance was not considered (unweighted UniFrac, *p* = 0.04), but not when it was considered (weighted UniFrac, *p* = 0.08) (Figure 4). This indicates that co-located horses shared some unique ASV, relative to the other horses, that were only present at low relative abundance. No ASVs or genera were significantly associated with FFW or control horses (*p* < 0.05; data not shown).

### 3.2. Assessment of the Effect of FMT on Horses Suffering from FFW

Compliance in the clinical field study was overall excellent, with the exception that the follow-up telephone-based questionnaire was not completed for two of the horses (P6 and P7). Throughout the study (d –9 to d 168), the lifestyle, training and feeding management of all the horses in the study only underwent subtle changes.

#### 3.2.1. FFW Symptom Severity and BCS

**Figure 5:**
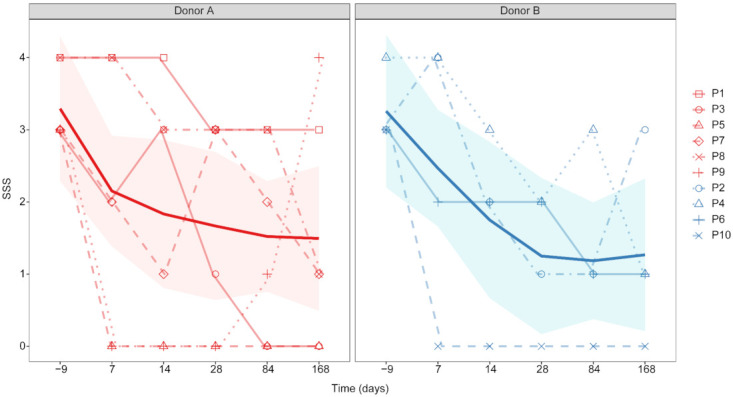
Plots showing the symptom severity scale (SSS) grades of the FFW horses (P1 to P10) in the study before (d –9) and after FMT (d 7, d 14, d 28, d 84 and d 168) with donor inocula prepared from either animal A or B. The bold line in each plot shows the median SSS grade for the FFW horses treated by the same donor.

All of the FFW horses exhibited severe symptoms (i.e., SSS 3 or 4) before receiving the standardized FMT protocol. The FFW horses varied in terms of presentation (i.e., ability to form faecal balls) and recognized triggers (Table 2). All FFW horses underwent the treatment without any adverse responses to the standardized FMT protocol. The response to the FMT treatment varied greatly among the FFW horses, with some having complete resolution and others only a partial decrease of symptom severity (Figure 5). At d 7, three horses had complete resolution of FFW symptoms (SSS of 0), however, between d 84 and d 168 one of these three horses had a relapse (Figure 5).

As choice of donor had no significant effect on SSS grades at any point in the study (*p* < 0.05), the data for all ten horses was analysed together. Sampling day had a significant effect on SSS grade (*p* = 0.028). Compared to d –9 (mean 3.3, SEM 0.15), the SSS grades were significantly lower in the FFW horses by d 14 (mean 1.8, SEM 0.46; *p* = 0.02), and then remained significantly lower until their last assessment on d 168 (mean 1.4, SEM 0.45; *p* < 0.02). BCS was not affected by time in FFW horses during d –9 to d 168 (*p* = 0.369; Table S4). Change in BCS relative to d –9 was also not affected by time in FFW horses during d 7 to d168 (*p* = 0.281; Table S4).

According to the eight completed questionnaires for the FFW horses at d 336, seven FFW horses had experienced a relapse of FFW symptoms to some extent, and one horse (P4) had been euthanised due to severe colic (Table 2).

#### 3.2.2. Donor Inocula and FFW Horse Faecal Microbiota

**Figure 6:**
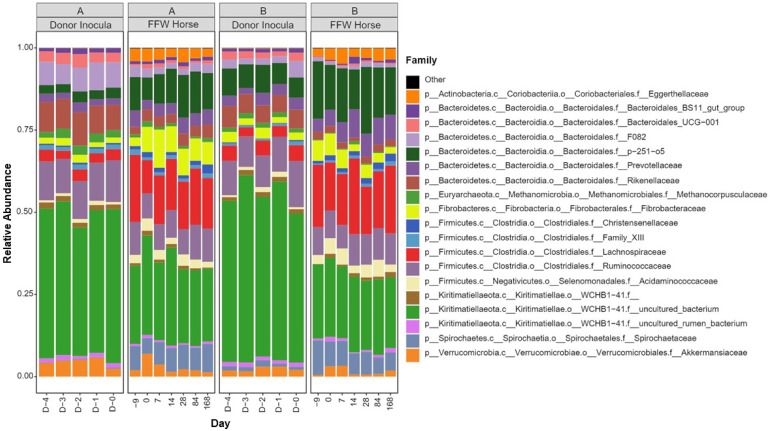
Taxonomic summary of the donor inocula and FFW horse faecal microbiota samples at the family level. For both donor animals (i.e., A and B), average profiles are shown per donor animal for the donor inoculant that was prepared on the five consecutive days (i.e., D-4 to D0). Average profiles for faecal microbiota of the FFW horses over the sampling time course (i.e., day –9 to day 168) are shown grouped based on the donor animal that was used during their FMT. The taxa listed in the key are shown in the order that they are appear within the plot.

The average number of reads per faecal sample was 115825 with a standard deviation of 53976. Family level taxonomic summaries of the prokaryotic community composition of the donor inocula and the FFW horse faecal microbiota are shown (Figure 6). Differences between the donor inocula and the FFW horse faecal microbiota can be clearly seen. For example, inocula from both donor animals had decreased relative abundances of Lachnospiraceae and Spirochaetaceae compared to the FFW horse faecal microbiota samples, irrespective of sampling day. No statistical analysis was performed of this, however, as the faecal material used to make the donor inocula was not sequenced.

Alpha diversity of the donor inocula samples was significantly lower (*p* < 0.001) than that of the FFW horse faecal microbiota samples with all four of the metrics that were used (Figures S2–S5). In contrast, no effect of sampling time on the alpha diversity of the FFW horse faecal microbiota samples was detected by any of the four metrics (Figures S2–S5).

**Figure 7:**
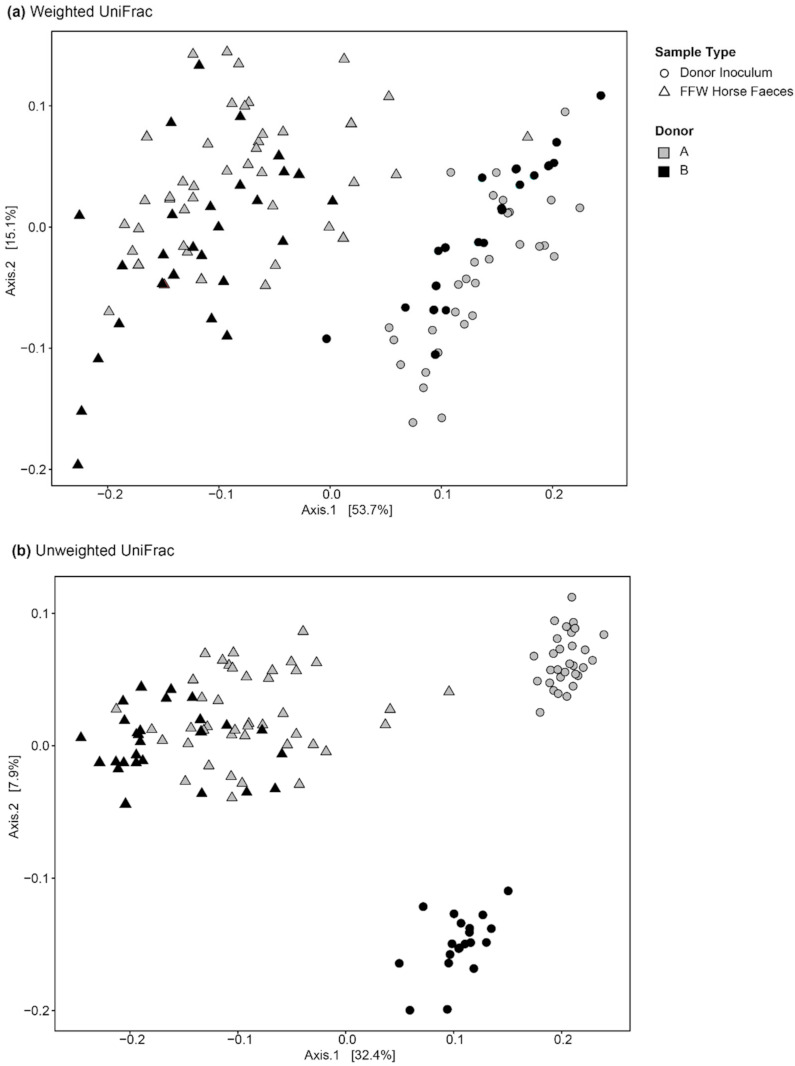
PCoA plots of 16S rRNA gene-based prokaryotic community composition data from faecal microbiota of the FFW horses (before and after FMT, i.e., seven samples per FFW horse) and the donor inocula they were treated with (i.e., five samples per FFW horse). Analysis was performed with both weighted (**a**) and unweighted (**b**) UniFrac distances. In the plots, the shape of the symbols indicates whether the sample is donor inoculum or FFW horse faeces. The symbol colour indicates the donor animal that the sample was associated with (i.e., which animal the inoculum was prepared from or, in the case of the FFW horse faeces, the animal that received it).

In terms of beta diversity, weighted PCoA indicated no clear differences existed between the donor inocula from the two different donor animals (Figure 7a). However, with unweighted PCoA the donor inocula from the two donor animals clearly separated (Figure 7b). This indicates that differences between the inocula from the two different donors were mainly due to unique ASVs that were of low relative abundance.

**Figure 8:**
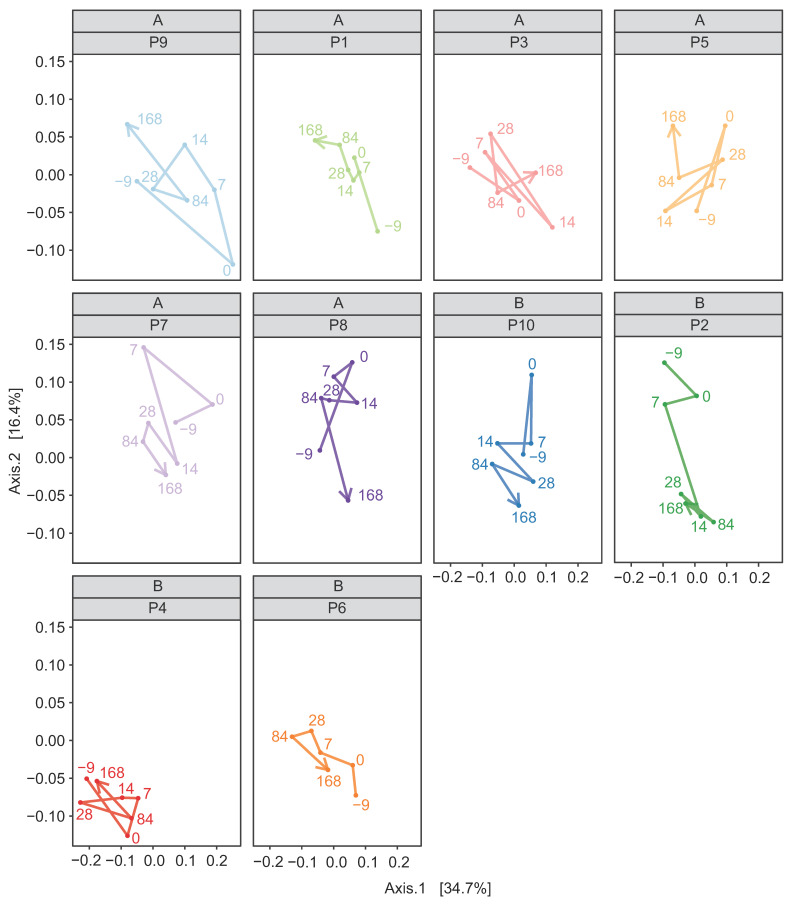
Weighted UniFrac PCoA of 16S rRNA gene-based prokaryotic community composition of faecal microbiota from individual FFW horses (i.e., P1 to P10). Samples from before (d –9) and after FMT (d 0, d 7, d 14, d 28, d 84 and d 168) are shown (i.e., seven samples for each FFW horse). The time course through the samples is indicated by a line, the direction of which is indicated by the arrowhead. The identity of the donor animal used to prepare the inoculant for each FFW horse is indicated above the FFW horse identifier.

**Figure 9:**
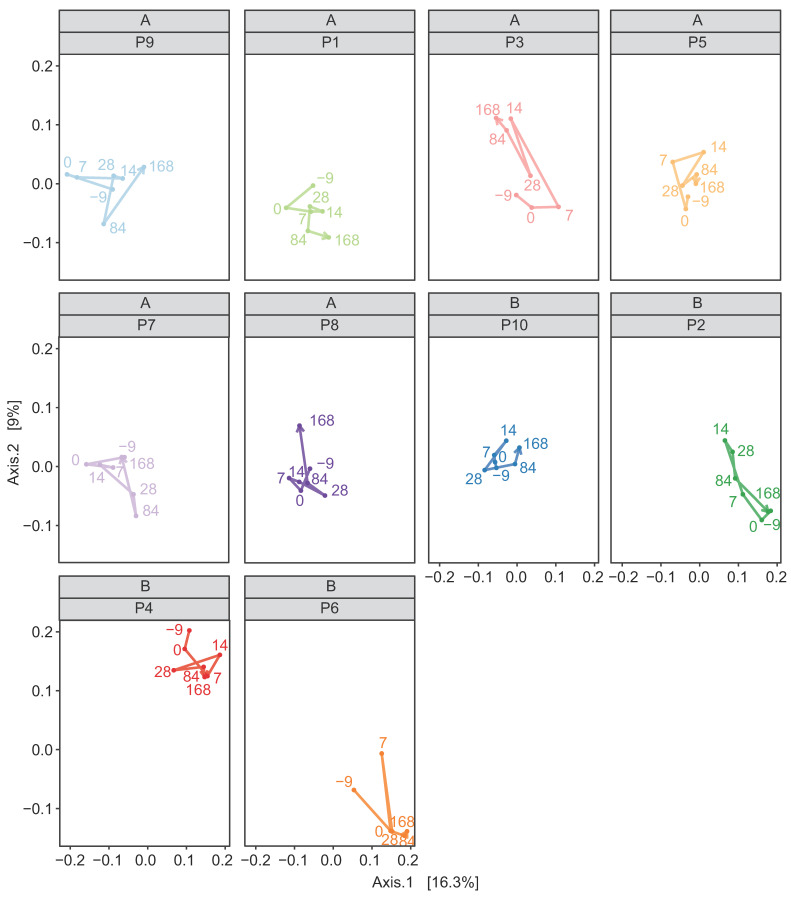
Unweighted UniFrac PCoA of 16S rRNA gene-based prokaryotic community composition of faecal microbiota from individual FFW horses (i.e., P1 to P10). Samples from before (d –9) and after FMT (d 0, d 7, d 14, d 28, d 84 and d 168) are shown (i.e., seven samples for each FFW horse). The time course through the samples is indicated by a line, the direction of which is indicated by the arrowhead. The identity of the donor animal used to prepare the inoculant for each FFW horse is indicated above the FFW horse identifier.

**Figure 10:**
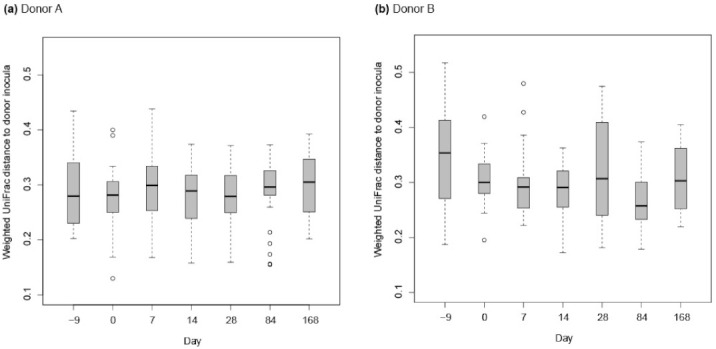
Boxplots are shown by donor animal (i.e., A or B) for the similarity of FFW horse faecal microbiota with the donor inocula over time using weighted UniFrac. All pairwise distances of a FFW horse faecal microbiota sample at one interval with all donor inoculant samples was used to generate the box plots (e.g., FFW horse faecal microbiota sample d –9 was compared with donor inoculant samples D-4, D-3, D-2, D-1, D0; FFW horse faecal microbiota sample d0 was then compared with donor inoculant samples D-4, D-3, D-2, D-1, D0 etc.). Boxes show the 25th and 75th percentiles with the median represented by a horizontal line. Whiskers show the data range with the exception of outliers that are indicated by data points.

**Figure 11:**
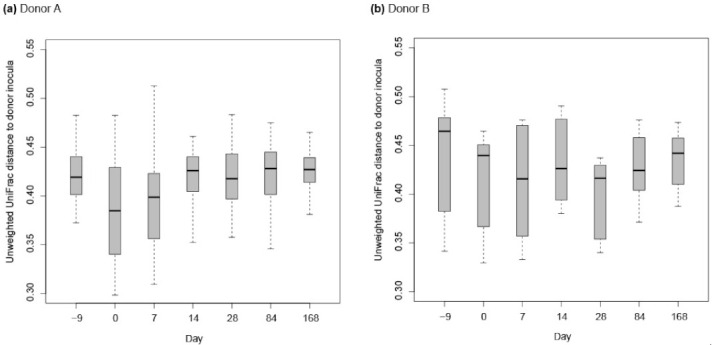
Boxplots are shown by donor animal (i.e., A or B) for the similarity of FFW horse faecal microbiota with the donor inocula over time using unweighted UniFrac. All pairwise distances of a FFW horse faecal microbiota sample at one interval with all donor inoculant samples was used to generate the box plots (e.g., FFW horse faecal microbiota sample d –9 was compared with donor inoculant samples D-4, D-3, D-2, D-1, D0; FFW horse faecal microbiota sample d 0 was then compared with donor inoculant samples D-4, D-3, D-2, D-1, D0 etc.). Boxes show the 25th and 75th percentiles with the median represented by a horizontal line. Whiskers show the data range.

In both the weighted and unweighted PCoA, the FFW horse faecal microbiota samples were separated from the donor inocula with the exception of one d 0 sample in the weighted PCoA (Figure 7a). There was also no consistent separation of the FFW horse faecal microbiota samples relative to before/after FMT, or time after FMT, when individual FFW horses were plotted using either weighted (Figure 8) or unweighted PCoA (Figure 9). Comparison of the similarity of the FFW horse faecal microbiota and the corresponding donor inocula samples over time also showed no significant differences (*p* > 0.05) when using either weighted (Figure 10) or unweighted UniFrac distances (Figure 11).

## 4. Discussion

The cause of FFW in equines is not known and was hypothesized in this study to involve the gut microbiota. Analyses in this study showed no significant differences in faecal microbiota of the FFW and control horses in terms of alpha or beta diversity. This finding verified the findings of two previous studies that had methodological limitations (i.e., delayed sample storage after collection and no Kiritimatiellaeota detected) [2,5]. In this study, the Kiritimatiellaeota phylum was found in all the equine faecal samples analysed, as has been previously reported [15,16]. Within the sampling time points, two previous studies reported that some minor taxa significantly differed in FFW horses relative to healthy horses [5,14]. In this study, no taxa were found that significantly differed between the control and FFW horses. It is not clear if this is due to methodological differences or the smaller number of animals used (*n* = 10) compared to the other two studies (i.e., *n* = 15 [5], *n* = 50 [14]). While FFW microbiota studies to date have analysed faecal microbiota, it is possible that faeces are not representative of the gut site most affected by the condition. Therefore, future microbial analysis of FFW horses should also consider analysis of mucosal biopsies and/or the free faecal water itself.

Differences in the diet and management of the horses may also have caused variation that masked more subtle differences in the faecal microbiota associated with FFW. For example, McKinney et al. [13] reported that the location of horses had a significant impact on their faecal microbiota. However, due to the isolated nature of the FFW cases in this study it was not possible to standardize the location, diet or management of all the horses. However, for each FFW horse a healthy control horse was recruited from the same location. This co-location approach allowed detection of low abundance ASVs that were unique to co-located horses, and presumably were associated with the local environment/diet/management of the horses.

Despite the lack of differences in the faecal microbiota of the FFW and control horses, the standardized FMT protocol used in this study significantly decreased FFW symptom severity. The onset of decreased symptom severity was not significant until 14 days after the FMT, although 30% of the FFW horses experienced complete resolution of symptoms after 7 days. The reason for the substantial variations in response to the FMT is not clear. Pre-treatment symptom duration and individual variation in triggering factors may have been contributing factors. As with studies on IBS in humans [39], it may also be important to subtype the phenotype of horses suffering from FFW as they may represent different etiologies of the condition. For example, such differences could include whether the FFW is constant, or if episodes are triggered by identifiable factors, and whether animals can form regular faecal balls.

Unlike previously reported [1], the majority of the FFW horses in this study failed to form regular faecal balls. Abnormalities in gut motility may result in the production of faecal water, as has been previously reported in one case where diarrhoea also occurred [40]. Diarrhoea is a clinical condition where uncontrolled release of faecal material is normally explosive and associated with a spasmodic episode, with abdominal discomfort/pain and ultimately dehydration. This is not characteristic of FFW or any of the FFW horses recruited to this study. Peristalsis and segmentation in the colon transversum leads to separation of the luminal solid content from the liquid, and the characteristic formation of faecal balls [41]. It has been suggested subtle changes in peristalsis, such as less haustral and more phasic or stronger phasic contractions, might contribute to FFW formation [1].

Monitoring changes in the faecal microbiota of FMT treated horses may provide further insight into the basis of their conditions. For example, McKinney et al. [13] reported that improving diarrhoea scores were associated with the faecal microbiota of the FMT recipient becoming more like the donor in terms of an increasing Verrucomicrobia abundance and alpha diversity. However, as no control animals were included in the study, the findings should be interpreted cautiously. In this study, the FMT did not affect the faecal microbiota alpha or beta diversity of FFW horses directly after the last transplantation was performed. These findings are difficult to compare to the study of McKinney et al. [13] as their reported analysis was limited to during and one day after FMT. Also, unlike diarrheic horses, no gut microbiota disruption or difference in the alpha or beta-diversity of the FFW horses was evident relative to healthy controls. The lack of change in the faecal microbiota of the FFW horses after d 0 was also true of FFW horses P5 and P10, which were clear responders to the FMT.

It should be noted that faecal microbiota transplants do not solely contain microbes, but also metabolites. It cannot be ruled out in this study that the alleviation of FFW symptom severity was influenced by the effects of metabolites provided in the donor inoculum. For example, volatile fatty acids are abundant metabolites in faeces, and of these, butyric acid is well-known for its ability to enhance gut integrity and development, minimize inflammation and alleviate ‘leaky gut’ related issues [42,43,44]. As well as microbial metabolites in the donor inoculum itself, it has been reported that with IBS patients the microbe-metabolite interactions seem to be disrupted after FMT [21]. Based on this, it is recommended that future equine FMT studies also include analysis of the metabolites in the donor inocula and the recipient’s faeces.

Of course, the donor inoculum itself was only one part of the standardized FMT protocol used in this study. A full dose of the proton-pump inhibitor (PPI) omeprazole was administered as well as psyllium. Omeprazole decreases gastric acid production in parietal cells and raises the gastric pH > 4 for up to 10 h after administration [45,46,47]. PPIs have been recommended when performing FMT as the acidic gastric environment can limit the viability of the transplanted microbiota [27,48]. Psyllium is a dietary fibre that has a gut-stimulatory effect, and can be used in the treatment of diarrhoea, constipation and sand accumulation [49,50,51]. Whilst it could be speculated that omeprazole and/or psyllium directly contributed to the decreased FFW symptom severity observed in this study, this would seem unlikely as no significant decrease in FFW symptom severity was seen until 14 days after their last administration. Furthermore, treatment with adsorptive supplements for all ten of the FFW horses was previously unsuccessful, and one horse had also been previously treated with omeprazole. However, in order to verify this, in future studies a control group of FFW horses only receiving the combined administration of omeprazole and psyllium should be included. Furthermore, treatment of the control group also with 5 L of saline for 5 days should be considered if the effect of the microbiota in the donor inoculum itself is to be directly assessed. This is due to the acidic pH of saline. Future studies may also consider using a different medium for preparing the donor inoculum that has a more neutral pH.

In addition to the lack of a control group, this study had some other limitations. The number of FFW horses used was only ten, which as mentioned earlier in the discussion is lower than previous studies with FFW horses [5,14]. Future longitudinal studies should use more FFW horses, particularly when FFW triggers within the cohort are more seasonal in nature. The donor animal faecal samples used to prepare the inoculum in this study were not analysed, which meant the effect of the inoculum preparation method relative to the original faecal sample it was derived from could not be directly assessed. As the findings of this study suggest that donor inoculum differs from faecal microbiota in terms of its composition and alpha diversity, it is recommended in future FMT studies that the microbiota of the donor inoculum is also assessed. This is particularly true if different inoculum preparation parameters are being tested. Whilst some insights can be gained about preferable inoculum preparation and storage methods using in vitro systems [48], the optimization of parameters based on in vivo studies should always be the ultimate goal. However, optimization of FMT protocol parameters is only practical when patients are likely to be responsive to FMT. Future equine research, therefore, also needs to focus on identifying diseases that are responsive to FMT, as well as understanding why only some animals are responsive. Within ethical constraints, future equine clinical studies should also consider including monitoring of a comparable cohort of non-FMT treated diseased animals in order to give insight into the normal variation in symptom severity over time.

Differences exist between the standardized FMT protocol used in this study and that previously reported [13,25]. It is not clear if these differences are critical to the efficacy of FMT. Besides the amount and frequency of transplant administered, other differing factors include the use of fresh versus stored (−20 °C) donor inoculum and homogenization versus sieving of inoculum to remove large fibrous particulates (to prevent nasogastric tube blockage). These latter factors will not uniformly affect the microbiota in the transplant, as some bacteria are more sensitive to freeze/thaw than others [48], and beneficial fibre degrading microbes are enriched on fibrous particles [52,53].

In this study, an SSS was developed to aid the assessment of FFW symptom severity. This scale has some similarities to the human “Bristol Stool Scale” [54]. The SSS scale was easy and comprehensive to use when grading FFW severity. As such, it is also a useful and practical tool to help veterinarians and/or owners identify potential FFW triggers and/or the impact of treatment interventions. In this study, six of the FFW horses were reported to have less severe symptoms during the summer and/or symptoms worsening in cold weather, whereas two horses had dietary triggers and the last two had constant FFW symptoms. The relative proportions of these perceived triggers differ from that previously reported, however, this may be due to the lower number of FFW horses in this study (*n* = 10) relative to that of the previous study (*n* = 42) [1].

## 5. Conclusions

In this study, it was found that horses suffering from FFW had no differences in their faecal microbiota compared to healthy controls. Using a standardized FMT protocol, FFW symptom severity was significantly reduced after horses received FMT. These findings indicate that while no hindgut microbiota disruption exists in FFW horses, FMT can potentially help to temporarily alleviate FFW symptom severity. However, these benefits could not be associated with any long terms change in the faecal microbiota following FMT. Due to differences in individual responses to FMT, additional studies using more animals and untreated FFW horses as controls are needed to gain further insight into (a) how best to identify FFW horses that are responsive to FMT and (b) how FFW symptom severity changes over time. Furthermore, analysis of faecal metabolites, as well as the microbiota, in future studies will advance fundamental understanding of how FMT influences the equine hindgut.

## Figures and Tables

**Table 1:** Standardised faecal microbiota transplantation (FMT) protocol used in this study.

Step	Description
Decrease gastric pH	Omeprazole 4 mg/kg BW SID (Equinor, ScanVet Animal Health, Fredensborg, Denmark) was given daily for ten days. Omeprazole was given in the morning, prior to the horses’ first meal. During the last five days of omeprazole treatment, the FMT was administered.
Preparation of FMT inoculum	Donor animals were selected in this study based on numerous criteria which, in no particular order, included: clinically healthy, no history of gut mediated disease, no medical treatment (other than standard anthelminthics) within the 12 months prior to being used as a donor, known clinical history (>5 years) and stabled in close proximity to the clinic. The donor faeces was collected daily by rectal grab sampling and was directly used to make the fresh FMT inoculum. Donor faeces (500 g) was sampled from the rectum of the donor horse. Donor faeces were placed into a bucket and gently mixed with 5 L of non-sterile saline that had been prewarmed to 37 °C. The inoculum was then passed through a sieve (mesh size approx. 5 mm) to remove large particles.
Administration of FMT	After five days of omeprazole treatment, administration of the FMT was started. FMT administration was repeated on five consecutive days (during which time omeprazole was still used) as follows: ○ Freshly prepared inoculum (5 L) was transplanted via a nasogastric tube. ○ Immediately after the inoculum was administered, a dose of psyllium 1 g/kg BW (Equiline, Loppefrøskaller, Provet, Kolding, Denmark) diluted in 5 L of tap water was administered via the nasogastric tube. If the horse showed signs of discomfort during administration, then the volume of psyllium solution was decreased so that a minimum psyllium dose of 0.5 g/kg bodyweight was administered. If required, horses were sedated prior to administration of the inoculum with detomidine 0.01 mg/kg BW (Cepesedan, ScanVet Animal Health, Fredensborg, Denmark).

**Table 2:** Details of the free faecal water (FFW) horses ^+^ in terms of the duration/onset of the issue, initial evaluation notes, donor animal used and the outcome of the faecal microbial transplant (FMT) treatment.

Horse Code	Duration/Onset of FFW Issue	Notes from Initial Evaluation for Study Recruitment	Donor Used	Status at d168	Follow Up at d336
P1	>1 year	Constant FFW with a lot of faecal water produced and no regular faecal balls. Variation within a day, with sometimes regular faeces.	A	Limited improvement to faecal consistency. Diagnosed with Cushing during the study.	Limited improvement to faecal consistency. Very dependent on feeding management. Reacts to subtle feed changes.
P2	>1 year	Periodic episodes of FFW daily, and able to form regular faecal balls. Summer symptoms less severe. A lot of abdominal gas and flatulence. Strong faecal odor. Cribbing behaviour.	B	Substantial improvement for 3 months, and then a return of symptoms between d84 and d168.	Occasional return of symptoms to same degree as before FMT. Stress induced.
P3	>1 year—onset after purchase and relocation of the horse.	Constant FFW with no regular faecal balls. A lot of abdominal gas and flatulence. Strong faecal odor. Aggressive behavioral pattern. Rejected rider. Skin and hoof problems.	A	Complete resolution of FFW symptoms. Skin and hoof completely normal.	Only one occasion of return of symptoms that was grazing induced. Returned to complete resolution spontaneously.
P4	>2 years—onset after feeding with wet haylage.	Constant FFW with no regular faecal balls. Extended abdomen with a lot of abdominal gas and flatulence. Recurring colic every two weeks. Colic surgery 12 months ago and associated antibiotic treatment afterwards. Very thin.	B	Many days with normal faeces or soft faeces. No colic for 5 months. Less flatulence. Weight gain and improved body condition.	Euthanized due to severe colic. Postmortem examination showed fibrino adhesions among organs in the abdominal cavity.
P5	>1 year	Constant FFW with no regular faecal balls. Less severe in summer. Occasional normal faeces for short periods during the day. Hoof problems.	A	Complete resolution of FFW symptoms.	Only one occasion of return of symptoms, which was grazing induced. Returned to complete resolution spontaneously.
P6	>1 year	Constant FFW with no regular faecal balls. Less severe in summer. Occasional normal faeces for short periods during the day. A lot of abdominal gas and flatulence. Strong faecal odor. Recuring colics. Antibiotic treatment worsened FFW symptoms.	B	FFW symptoms much improved. Only very few days with mild symptoms. No colic was reported after receiving the FMT.	No owner response to d336 questionnaire.
P7	>3 years	Periodic episodes of FFW daily with no regular faecal balls. Worsens in autumn.	A	Symptoms much improved, with only a few days a month with FFW (which appeared to be associated with cold weather).	No owner response to d336 questionnaire.
P8	>1 year	Constant FFW during autumn and winter. No regular faecal balls formed. Less severe in summer when grazing.	A	Symptoms improved only a little after FMT (which was performed in the winter).	Return of symptoms to same degree as before FMT. Induced by feeding of conserved forage (i.e., either hay or haylage).
P9	>6 years—onset at weaning	Constant FFW with no regular faecal balls. Worse while grazing.	A	Complete resolution for three months but return of constant FFW symptoms when grazed.	Return of symptoms to same degree as before FMT. Grazing induced.
P10	>3 years—onset at weaning	Constant FFW with no regular faecal balls. Worse in cold weather.	B	Complete resolution of FFW symptoms.	Occasional return of symptoms (associated with cold & damp weather) to a milder degree compared with before FMT.

^+^ All FFW horses had a history of FFW for >12 months, and no resolution despite various treatments. A diagnostic work-up was performed by the horse’s own veterinarian, the details of this and previous treatments are provided in Table S1.

## Data Availability

The raw sequence data for the first part of the study where samples from both FFW and control horses were analysed has been deposited in ENA under study accession number PRJEB35172. The raw sequence data for the longitudinal analysis of the FFW horses and the associated donor inocula they received has been deposited in ENA under study accession number PRJEB45364.

## Supplementary Materials

The following are available online at https://www.mdpi.com/article/10.3390/ani11102776/s1. Table S1—Summary of diagnostic analysis by the FFW horse’s own veterinarian, previous FFW treatments and travel time to the clinic for FMT; Table S2—Details of all the free faecal water, control, and faecal donor horses used in the study; Table S3—Phylum composition of the faecal microbiota of control and FFW horses; Table S4—Body condition score of the FFW horses at different days (d) throughout the study; Figure S1—Procrustes analysis plots of faecal prokaryotic community composition data from FFW and control horses using weighted and unweighted UniFrac distances; Figure S2—Alpha diversity values calculated using the Phylogenetic Diversity metric for donor inocula and FFW horse faecal microbiota samples; Figure S3—Alpha diversity values calculated using the InvSimpson metric for donor inocula and FFW horse faecal microbiota samples; Figure S4—Alpha diversity values calculated using the Richness metric for donor inocula and FFW horse faecal microbiota samples; Figure S5—Alpha diversity values calculated using the Shannon metric for donor inocula and FFW horse faecal microbiota samples.
