# Supplementary material for: Free Faecal Water: Analysis of Horse Faecal Microbiota and the Impact of Faecal Microbial Transplantation on Symptom Severity
Source: Animals (Basel). 2021 Sep 23;11(10):2776. doi: 10.3390/ani11102776 (PMC8533009; doi:10.3390/ani11102776)
Supplement: Supplementary file 1 [file animals-11-02776-s001.zip › animals-1384383-supplementary.pdf]

Supplementary Material

Free Faecal Water: Analysis of Horse Faecal Microbiota and the Impact of Faecal Microbial Transplantation on Symptom Severity

Louise Laustsen [1], Joan E. Edwards [2*], Gerben D.A. Hermes [2], Nanna Lúthersson [3], David A. van Doorn [4,5], Supattra Okrathok [2,6], Theresa J. Kujawa [5,7] and Hauke Smidt [2*]

[1] Department of Veterinary Education, Institute of Veterinary Science, University of Liverpool, Neston, CH64 7TE, UK; vetlouiselaustsen@gmail.com

[2] Laboratory of Microbiology, Wageningen University & Research, Wageningen, The Netherlands; gerben.hermes@wur.nl (G.D.A.H.); s.okrathok@gmail.com (S.O.)

[3] Hestedoktoren, Kirke Eskilstrup, Denmark; nanna@hestedoktoren.dk

[4] Department of Clinical Sciences, Equine Health, Utrecht University, Utrecht, The Netherlands; d.a.vandoorn1@uu.nl

[5] Department of Population Health Science, Farm Animal Health, Utrecht University, Utrecht, The Netherlands; theresa.k2k@gmail.com

[6] School of Animal Technology and Innovation, Institute of Agricultural Technology, Suranaree University of Technology, Nakhon Ratchasima 30000, Thailand

[7] Animal Nutrition Group, Wageningen University & Research, Wageningen, The Netherlands.

* Correspondence: joanee2002@hotmail.com (J.E.E.); hauke.smidt@wur.nl (H.S.)

Table S1. Summary of diagnostic analysis by the FFW horse's own veterinarian, previous FFW treatments and travel time to the clinic for FMT.

	Diagnostic Analysis							Previous FFW Treatments					
Patient Code	Abdominal Ultrasound	Abdominal Explorative Surgery	Gastroscopy	Rectal Biopsy	Rectal Exam	Blood Analysis	Parasites	Dietary Change	Antibiotics	Steroids	Absorptive Supplements	Other Treatments[#]	Travel Time to Clinic for FMT (hours)
P1	N	N	N	N	Y	Y	Y	Y	N	N	Y	-	1.0
P2	Y	N	N	N	Y	Y	Y	Y	N	N	Y	NSAID due to hoof problem/pain	2.5
P3	Y	N	Y	N	Y	Y	Y	Y	N	N	Y	NSAIDS for colic, omeprazol for EGUS and antibiotics after surgery	2.5
P4	Y	Y	Y	N	Y	Y	Y	Y	Y	Y	Y	-	2.5
P5	N	N	N	N	Y	Y	Y	Y	N	N	Y	NSAID due to lameness	0.5
P6	Y	N	Y	N	Y	Y	Y	Y	Y	N	Y	-	1.0
P7	N	N	N	N	Y	Y	Y	Y	NK*	N	Y	-	1.0
P8	N	N	N	N	Y	Y	Y	Y	N	N	Y	-	1.0
P9	N	N	N	N	Y	Y	Y	Y	N	N	Y	-	0.5
P10	N	N	N	N	Y	Y	Y	Y	N	N	Y	-	1.0

*NK, not known

NSAID, non-steroidal anti-inflammatory drug; EGUS, equine gastric ulcer syndrome

Table S2. Details of all the free faecal water (P), control (C), and faecal donor (D) horses used in the study.

Horse Code	Age (years)	Type[#]	Sex	Purpose*	Forage	Concentrate kg/day	Bedding[$]	Herd Hierarchy[+]	Turnout^ (hours/day)
P1	21	WB Horse	Gelding	Recreational	Haylage	0.85	Straw	Medium	8
C1	10	WB Horse	Mare	Breeding	Haylage	1.50	Straw	High	8
P2	8	Sports Pony	Gelding	HI Dressage	Haylage	1.70	Straw	Medium	2
C2	10	Sports Pony	Gelding	HI Dressage	Haylage	0.30	Straw	Medium	2
P3	9	Sports Pony	Gelding	Dressage	Hay	0.70	Straw	Medium	0
C3	6	Sports Pony	Mare	Dressage	Hay	0.15	Straw	Medium	0
P4	8	WB Horse	Gelding	Dressage	Hay	2.45	Shavings	Medium	7
C4	17	WB Horse	Gelding	Dressage	Hay	1.80	Straw	High	7
P5	7	WB Horse	Gelding	Dressage	Hay	0.53	Shavings	Medium	8
C5	10	WB Horse	Gelding	Dressage	Haylage	No Data	Shavings	Medium	8
P6	18	WB Horse	Gelding	Dressage	Hay	1.20	Shavings	High	8
C6	17	WB Horse	Stallion	Dressage	Hay	1.20	Shavings	High	8
P7	7	CB pony	Gelding	Pleasure	Haylage	1.20	Straw	Medium	7
C7	10	Sports Pony	Gelding	Pleasure	Haylage	0.30	Straw	Medium	7
P8	6	WB Horse	Gelding	Eventing/Show jumping	Haylage	0.65	Straw	Medium	10
C8	4	WB Horse	Gelding	Eventing/Show jumping	Haylage	1.20	Straw	Medium	10
P9	12	WB Horse	Mare	Dressage	Hay	1.80	Straw	Medium	5
C9	11	WB Horse	Mare	Dressage	Hay	1.80	Straw	Medium	5
P10	9	CB Pony	Mare	Carriage driving	Hay	0.45	Straw	Medium	12
C10	14	CB Pony	Gelding	Carriage driving	Hay	0.45	Straw	Medium	12

DA	17	WB Horse	Mare	Recreational	Haylage	1.00	Shavings	Medium	8
DB	15	WB Horse	Mare	Recreational	Haylage	1.00	Shavings	Low	8

WB, warmblood; CB, coldblood

* HI: high intensity training

$ All horses were individually boxed, and the type of bedding used is indicated.

+ Herd hierarchy is indicated for when the horses were turned out in a paddock with other horses.

^ Horses were turned out in a dirt paddock.

Table S3. Phylum composition of the faecal microbiota of control and FFW horses.

	Control		Patient	
**Phylum (%)**	**Average**	**SEM**	**Average**	**SEM**
Firmicutes	33.11	1.44	33.42	1.86
Bacteroidetes	24.20	1.58	25.90	1.14
Kiritimatiellaeota	16.78	1.78	18.58	1.89
Spirochaetes	14.51	1.30	13.37	1.67
Fibrobacteres	7.15	1.84	4.47	1.11
Actinobacteria	1.40	0.21	1.64	0.19
Euryarchaeota	1.37	0.43	1.07	0.28
Verrucomicrobia	0.57	0.15	0.64	0.19
Cyanobacteria	0.31	0.14	0.29	0.10
Proteobacteria	0.14	0.04	0.14	0.05
Tenericutes	0.11	0.06	0.00	0.00
Planctomycetes	0.05	0.03	0.03	0.02
Lentisphaerae	0.02	0.02	0.00	0.00
Not Annotated	0.29	0.12	0.46	0.19

Table S4. Body condition score (BCS) of the FFW horses at different days (d) throughout the study.

Horse Code	BCS						Change in BCS relative to d-9				
	d-9	d7	d14	d28	d84	d168	d7	d14	d28	d84	d168
P1	5.0	5.0	5.0	5.0	5.0	5.0	0.0	0.0	0.0	0.0	0.0
P2	5.5	5.5	5.5	5.5	5.0	5.0	0.0	0.0	0.0	-0.5	-0.5
P3	5.5	6.0	5.5	5.5	5.5	5.5	0.5	0.0	0.0	0.0	0.0
P4	3.5	3.5	4	4.5	5	5	0.0	0.5	1.0	1.5	1.5
P5	5.5	5.0	5.0	5.0	5.0	5.0	-0.5	-0.5	-0.5	-0.5	-0.5
P6	5.5	5.5	5.0	5.5	5.5	5.5	0.0	-0.5	0.0	0.0	0.0
P7	7.0	7.0	7.0	7.5	7.0	5.5	0.0	0.0	0.5	0.0	-1.5
P8	5.5	5.5	5	5.5	5.5	5.5	0.0	-0.5	0.0	0.0	0.0
P9	6.5	6.0	6.0	6.0	6.0	7.5	-0.5	-0.5	-0.5	-0.5	1.0
P10	6.0	5.5	5.5	5.5	7.0	7.5	-0.5	-0.5	-0.5	1.0	1.5
Mean	5.6	5.5	5.4	5.6	5.7	5.7	-0.1	-0.2	0.0	0.1	0.0
SEM	0.29	0.28	0.25	0.25	0.25	0.31	0.10	0.11	0.15	0.21	0.30

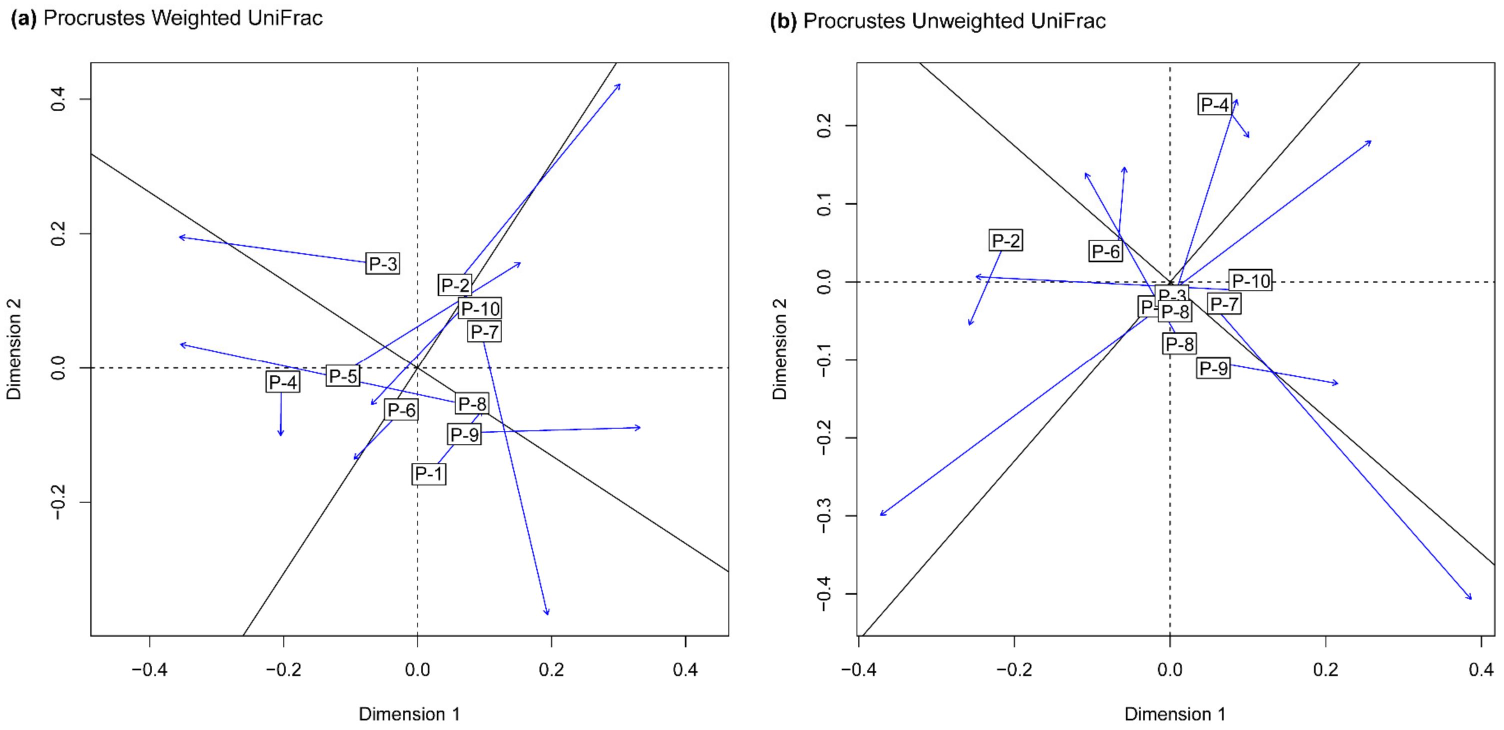

Figure S1. Procrustes analysis plots of faecal prokaryotic community composition data from FFW and control horses using weighted (**a**) and unweighted (**b**) UniFrac distances. Plots show a visual indication of the degree of match between the FFW and control horses ordinations. Labels show the position of the FFW horses ordination, and arrows point to their positions in the control horses ordination. The plot also shows the rotation between the two ordinations necessary to make them match as closely as possible.

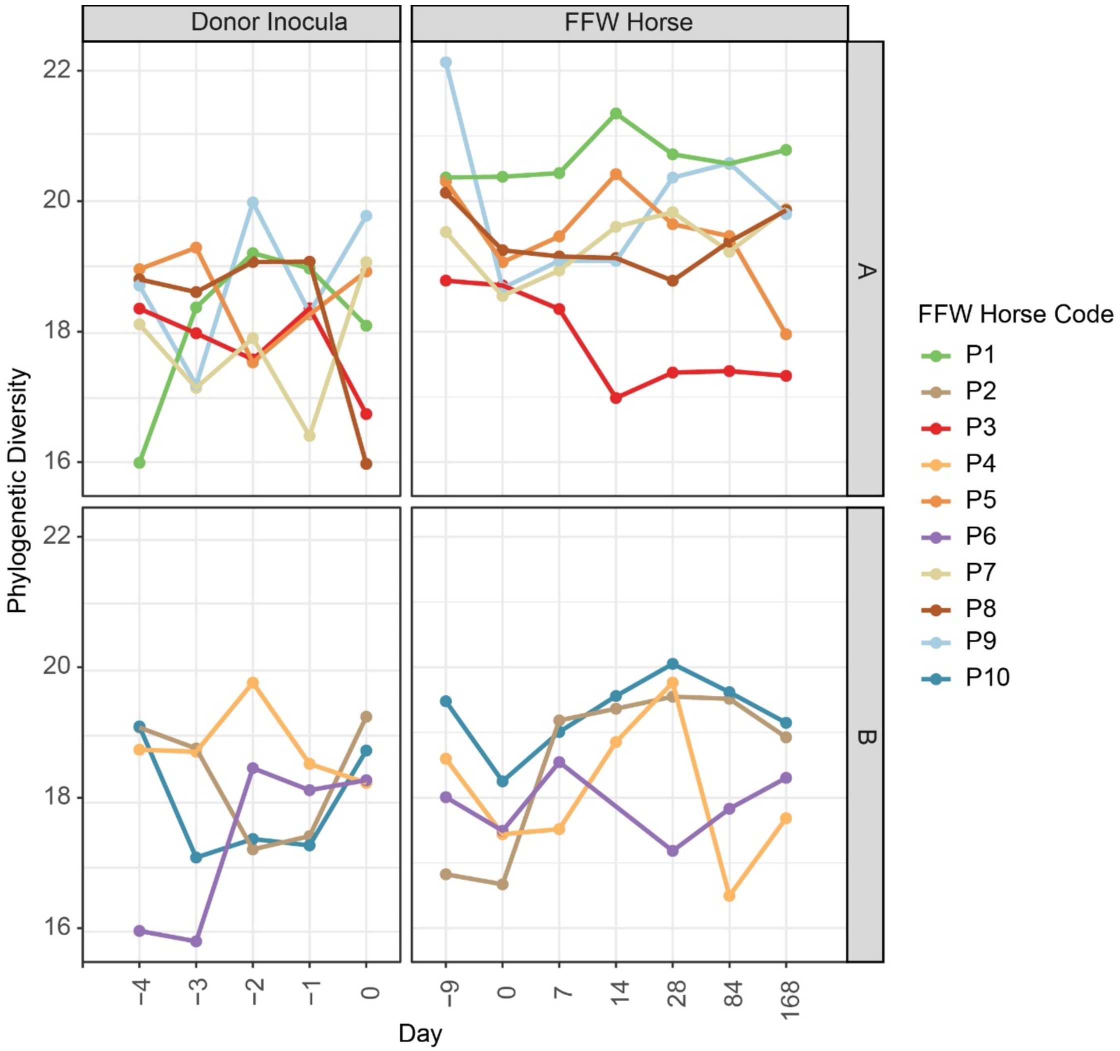

Figure S2. Alpha diversity values calculated using the Phylogenetic Diversity metric for donor inocula and FFW horse faecal microbiota samples. Donor inocula samples are colored by the animal that received them, and all FFW horse related samples are grouped by donor animal used (i.e., donor A or B).

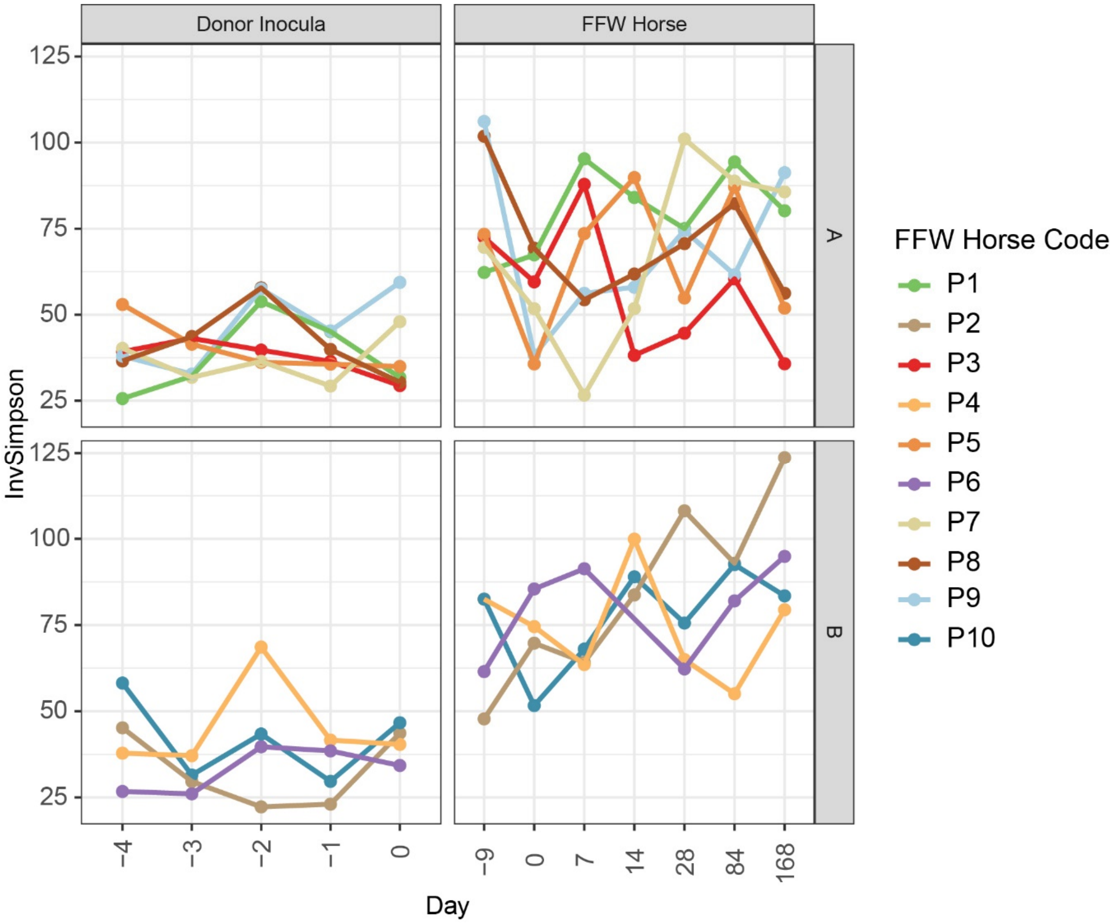

Figure S3. Alpha diversity values calculated using the InvSimpson metric for donor inocula and FFW horse faecal microbiota samples. Donor inocula samples are colored by the animal that received them, and all FFW horse related samples are grouped by donor animal used (i.e., donor A or B).

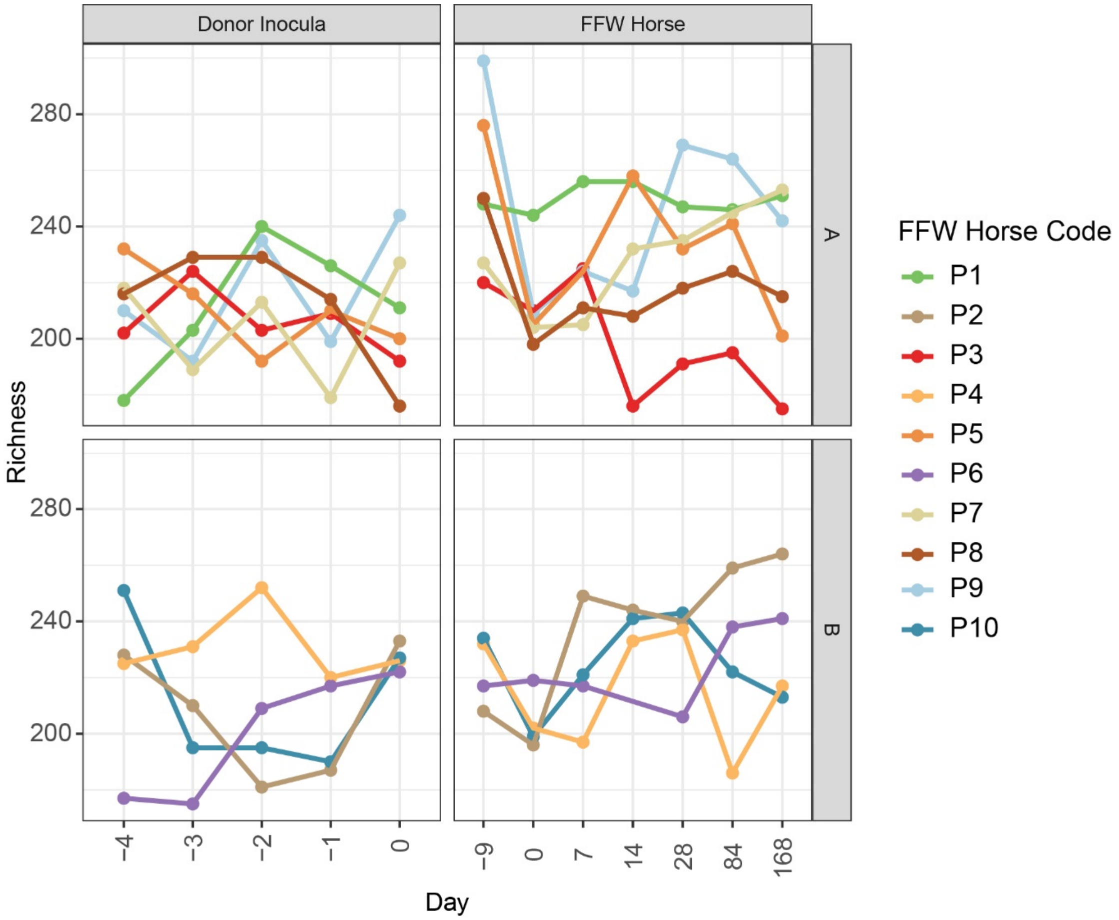

Figure S4. Alpha diversity values calculated using the Richness metric for donor inocula and FFW horse faecal microbiota samples. Donor inocula samples are colored by the animal that received them, and all FFW horse related samples are grouped by donor animal used (i.e., donor A or B).

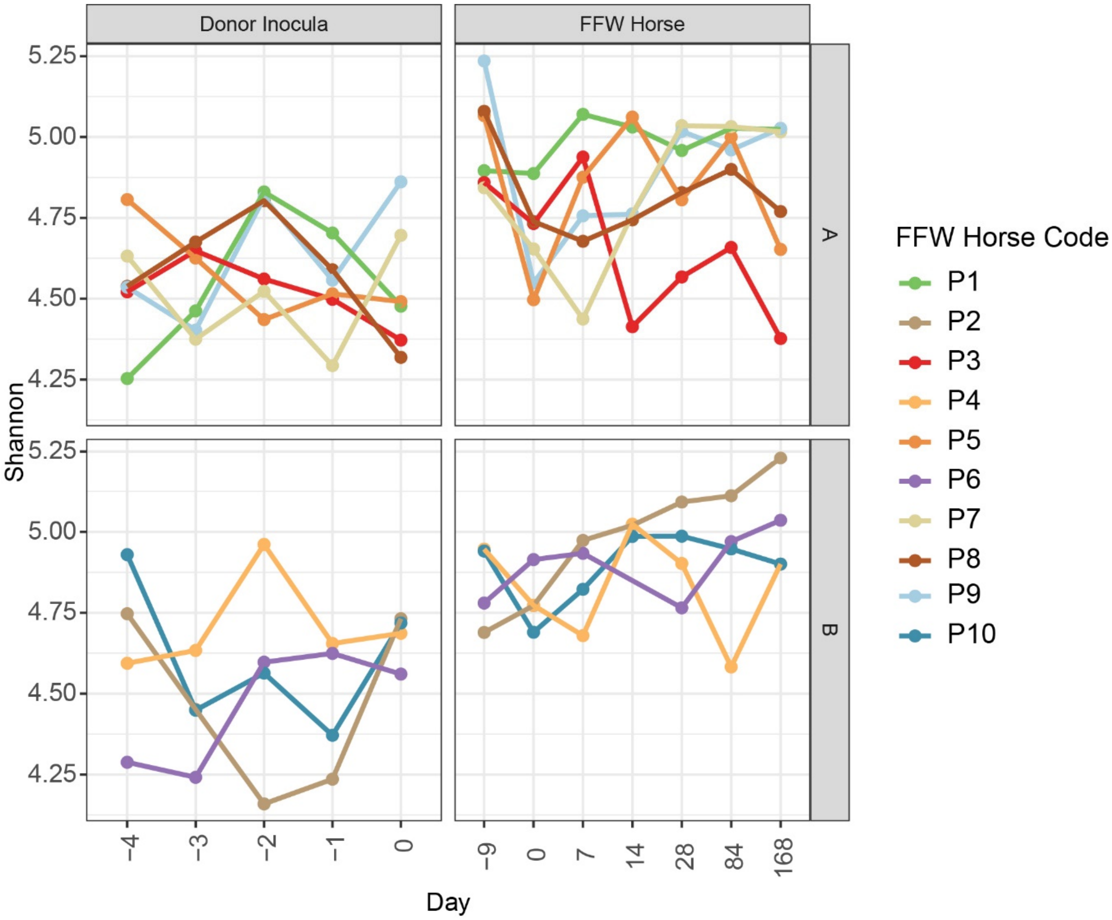

Figure S5. Alpha diversity values calculated using the Shannon metric for donor inocula and FFW horse faecal microbiota samples. Donor inocula samples are colored by the animal that received them, and all FFW horse related samples are grouped by donor animal used (i.e., donor A or B).
